# Mexico: A Landscape of Viroid Origin and Epidemiological Relevance of Endemic Species

**DOI:** 10.3390/cells11213487

**Published:** 2022-11-03

**Authors:** Katia Aviña-Padilla, Erika Janet Zamora-Macorra, Daniel Leobardo Ochoa-Martínez, Flor Citlally Alcántar-Aguirre, Maribel Hernández-Rosales, Loranda Calderón-Zamora, Rosemarie W. Hammond

**Affiliations:** 1 Centro de Investigacion y de Estudios Avanzados del I.P.N. Unidad Irapuato, Irapuato 36821, Mexico; 2 Department of Crop Sciences, University of Illinois at Urbana-Champaign, Urbana, IL 61801, USA; 3 Universidad Autonoma Chapingo, Km 38.5, Carretera Mexico-Texcoco, Chaping 56230, Mexico; 4 Colegio de Postgraduados, Posgrado en Fitosanidad-Fitopatología, Texcoco 56230, Mexico; 5 Instituto de Neurobiologia, Universidad Nacional Autonoma de México, Queretaro 76230, Mexico; 6 Facultad de Biologia, Universidad Autonoma de Sinaloa, Calzada de las Americas y calle Universitarios, s/n Ciudad Universitaria, Culiacan 80013, Mexico; 7 USDA, Agricultural Research Service, Beltsville Agricultural Research Center, Beltsville, MD 20705, USA

**Keywords:** origin and evolution, geographical distribution and epidemiology, subviral plant pathogens, viroid transmission, economic significance, viroids

## Abstract

Viroids are single-stranded, circular RNA molecules (234-406 nt) that infect a wide range of crop species and cause economic losses in agriculture worldwide. They are characterized by the existence of a population of sequence variants, attributed to the low fidelity of RNA polymerases involved in their transcription, resulting in high mutation rates. Therefore, these biological entities exist as *quasispecies*. This feature allows them to replicate within a wide range of host plants, both monocots and dicots. Viroid hosts include economically important crops such as tomato, citrus, and fruit trees such as peach and avocado. Given the high risk of introducing viroids to viroid disease-free countries, these pathogens have been quarantined globally. As discussed herein, Mexico represents a geographical landscape of viroids linked to their origin and comprises considerable biodiversity. The biological features of viroid species endemic to Mexico are highlighted in this communication. In addition, we report the phylogenetic relationships among viroid and viroid strains, their economic impact, geographical distribution, and epidemiological features, including a broad host range and possible long-distance, seed, or insect-mediated transmission. In summary, this review could be helpful for a better understanding of the biology of viroid diseases and future programs on control of movement and spread to avoid economic losses in agricultural industries.

## 1. A Landscape of Viroid Biodiversity in Mexico

Viroids were discovered by plant pathologist Dr. Theodor O. Diener while undergoing efforts to identify the cause of the potato spindle tuber disease. In the first instance, the disease was believed to be caused by a virus. Experiments designed to identify the hypothetical presumed virus yielded unexpected results. The unconventional particle was sensitive to treatment with ribonuclease (no infectivity) and insensitive to deoxyribonuclease, phenol, chloroform, n-butanol, or ethanol treatments. In 1967 it became evident that the agent of the potato spindle disease was not a virus, and the term *“viroid”* was proposed for this novel free infectious RNA.

Potato spindle tuber viroid (PSTVd) was the first viroid species discovered more than 50 years ago in the northeastern region of the United States [1,2,3,4]. Almost simultaneously, another significant viroid disease was described by Dr. Joseph Semancik, affecting citrus in that country [5,6].

Around 1972 a severe disease of tomato was observed in Mexico by plant virologist Dr. Jorge Galindo-Alonso in the central region, specifically in the states of Morelos and Mexico. The disease was locally named “*Planta macho*”(male plant) because one of the main identified symptoms was flower abortion [7]. At that time, the disease was also thought to be of virus origin. Later work using electron microscopy revealed the lack of virus particles [8]. In the following years, Galindo, Smith, and Diener performed a deep etiological study to confirm the viroid nature of the “*Planta Macho*” disease. The disease agent was named tomato planta macho viroid (TPMVd). Notably, in bioassays in infected tomato plants, TPMVd showed more severe symptoms compared to those of PSTVd [9].

Presumably, the origin of viroid crop diseases as an outcome of their initial presence in wild plant species reservoirs (symptomless) was considered by Galindo and Diener. Previous reports speculated that TPMVd was infecting tomato crop fields as a possible transfer by an aphid vector from endemically infected native species from the *Solanaceae* family [10].

Mexico is well known as a prominent center of the diversity and endemism of several *Solanaceae* members. At least 135 species are endemic to this country [11]. The awareness of this biodiversity and the fact that previous explorations in Peru to detect viroids in wild hosts were unfruitful prompted Diener and Galindo to perform an expedition searching for the possible origin of PSTVd in Mexican wild potato. During this expedition, Mexican papita viroid (MPVd), currently considered a genetic variant of TPMVd, was identified in symptomless *Solanum cardiophylum* plants. The asymptomatic host behavior, and the distribution of eight isolates in a defined geographical area, suggested a possible viroid/host *“coevolution”* process [12].

In the following years, the noticeable presence of viroid diseases affecting citrus in northeastern Mexico was reported to dramatically affect orange and lime groves [13,14,15,16]. Outbreaks of citrus viroid diseases became apparent from 2000 to 2018. Interestingly, the coexistence of the citrus tristeza virus (CTV) and a viroid species in orange trees acting in synergy was recently linked to strong symptom development [17].

Until 2009, no viroid species of the *Avsunviroidae* family were reported in Mexico. The first official report describing the presence of avocado sunblotch viroid (ASBVd) in avocados was from a primary producer region in Michoacan [18]. Later, in 2015, peach latent mosaic viroid (PLMVd) was identified in peach orchards in central Mexican states [19].

Like the characteristic endemism of plant species belonging to the *Solanaceae* family, the origin of the avocado plant has been determined to belong to a particular region of Mexico. In this context, it has recently been proposed that the origin of ASBVd resembles the origin of its natural host [20]. This proposal relies on the observation that the southern region of California imported seeds from Mexico at the beginning of the establishment of the avocado industry. It is possible that the germplasm could have been infected with ASBVd before the cross-border movement occurred. Hence, a similar ASBVd/avocado *“coevolution”* mechanism was postulated as previously reported for the MPVd/Mexican wild papita combination.

As discussed above, it is significant that Mexico represents a geographical landscape of viroid’s ancestral origin and comprises considerable biodiversity, including endemic species and mixed infections affecting agronomically important crops such as tomato and high-value products such as avocado. In this communication, we focus on the genomic and biological features of TPMVd and MPVd, their genetic variants, and those of ASBVd, all of which belong to viroid species considered endemic to Mexico. We also discuss the relevance of other tomato and citrus viroid disease species that have been reported in this country.

## 2. Mexican Viroid Species: Economic Impact, Disease Relevance, and Spread

**Figure 1:**
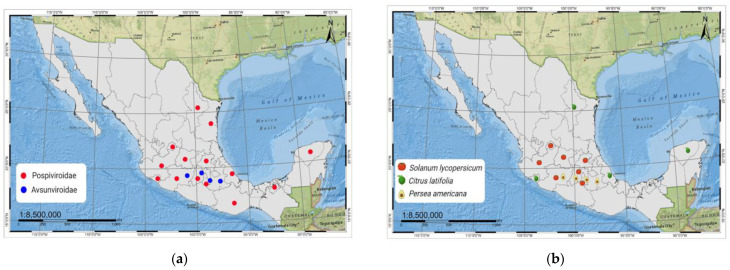
(**a**) Geographical distribution of the seven viroid species across Mexico; red dots depict the five viroid species that belong to the *Pospiviroidae* family, while the blue dots the two species in the *Avsunviroidae*. (**b**) Distribution of the affected economic important hosts due to viroid diseases across the country.

To date, more than 40 viroid species have been reported exclusively from angiosperms. In this context, 25 viroid diseases have been communicated worldwide in multiple crops, including vegetables, fruit trees, and ornamentals. Notably, 48 isolates from 7 viroid species belonging to the two known families have been officially reported in Mexico, as depicted in Figure 1 (https://www.ncbi.nlm.nih.gov/nuccore, accessed on 27 June 2022). A detailed description of their economic impact, mode of spread, and control strategy is shown in Table 1.

The most critical economic hosts affected are tomato under field and greenhouse conditions, citrus, and avocado groves, Figure 1b. These viroid diseases’ symptoms include dwarfing and leaf malformation, abnormal enlargement and discoloration of fruit and pericarp in fruit trees, and disruptions in the biosynthesis of secondary metabolites.

### 2.1. Pospiviroidae Species Reported in Mexico

**Figure 2:**
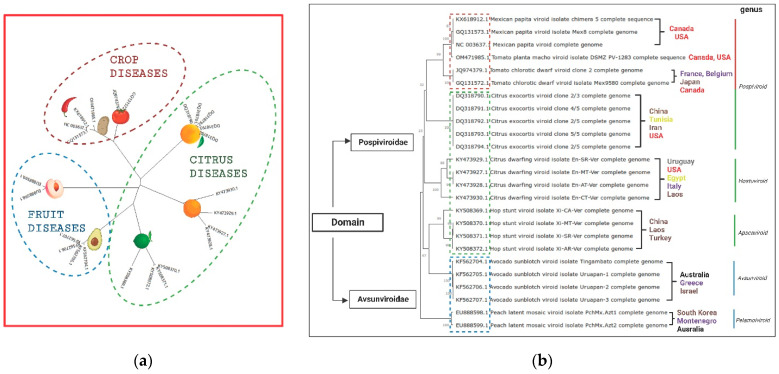
(**a**) A molecular phylogenetic tree of Mexican viroids isolates with complete genomes in the NCBI. Viroid isolates related to crop diseases are clustered in red, while those in citrus diseases are in green and fruit diseases in blue circles, respectively. (**b**) A phylogenetic tree reconstruction using the UPGMA (MEGA X software) with 1000 bootstrap replicates of nucleotide sequences of officially reported viroids in Mexico, their relationship with isolates from other countries are colored by the continent of distribution, red: North America; gray: South America; purple: Europe; yellow: Africa; brown: Asia; black: Oceania in *Pospiviroidae* and *Avsunviroidae* species.

Five viroid species in this family have been identified in Mexico. TPMVd (MPVd), tomato chlorotic dwarf viroid (TCDVd), and citrus exocortis viroid (CEVd) are members of the *Pospiviroid* genus. Pospiviroids are potentially risky to major crops such as potato, chili pepper, and tomato, Figure 2a. Meanwhile, hop stunt viroid (HSVd), and citrus dwarfing viroid (CDVd) are species belonging to the *Hostuviroid* and *Apscaviroid* genera, respectively, capable of affecting a large proportion of citrus groves, Figure 2a. A full description of the dissemination of Mexican viroid isolates is listed in Table 2.

Tomato is one of the world’s most widely grown greenhouse vegetables [51] and is one of the most profitable crops widely consumed for its various products. In 2018, 243,888,041 tons were harvested [52]. In Mexico, the production of this crop is an important activity in the social and economic sphere due to its foreign exchange earnings and job creation [53]. Diseases caused by viroids in tomato crops can cause severe economic losses [39]. A decade ago, one of the emerging diseases in tomato under greenhouse conditions in North America was associated with Mexican viroid isolates, Figure 2b. The presence of TCDVd and MPVd viroids in tomato under greenhouse conditions was remarkable in those years [38,39,54,55].

In contrast to MPVd, TCDVd causes chlorotic leaves and severe dwarfing of plants in greenhouse tomato crops. The first report of MPVd and TCDVd severe infections in tomato under greenhouse conditions was in 2008 in greenhouses near Mexico City [39]. Molecular analysis indicated samples contained a mixed infection of the two pospiviroids. In addition, this was the first report of MPVd Mexican isolates identified with a 93 to 94% nucleotide sequence identity to other isolates outside the country. Moreover, a TCDVd isolate previously identified in Arizona was reported in central Mexico, Figure 2b. Later, viroid infections in tomato cherry plants (*S. lycopersicum* var. cerasiforme= tomato cherry) were identified in high-tech greenhouses under continuous production conditions. Infected plants exhibited symptoms like those associated with viroids, primarily stunting, epinasty, and chlorosis of young leaves. Symptoms rapidly propagated to different greenhouses during that summer. Two types of variants of TCDVd were identified. One variant showed 91% nucleotide sequence identity, and the other 95% with European isolates [56]. The origin of TCDVd in these greenhouses remains unknown.

Viroids officially reported affecting citrus groves comprise the most abundant number of Mexican isolates in the NCBI database. Citrus viroids infect a large number of species, such as *Citrus paradisi* and *Citrus latifolia*, causing significant economic losses. These viroids induce disease only when they infect a sensitive host, and three of the seven viroid species are present in Mexico, namely CEVd, CDVd, and HSVd [57]. CEVd causes stunting, bark sloughing and cracking leaf epinasty, and cracks in the petiole [58]. The isolates from Mexico deposited in the NCBI have a percentage of homology between 99% to 91%, with isolates from Tunisia (Africa), China, Iran (Asia), and the USA, Figure 2b. Another viroid species related to citrus diseases is CDVd which produces the severe bark scaling characteristic of exocortis disease in trifoliate orange rootstocks [59]. Variants of this species have nucleotide sequence homologies ranging from 99% to 88%, with isolates from Uruguay, USA, Egypt, Italy, and Laos, and it is one of the most widely distributed species. Meanwhile, HSVd has also been identified in Mexico citrus groves with 100% to 87 homologies with other isolates such as Turkey, China, Italy, and Laos.

### 2.2. Avsunviroidae Species

**Figure 3:**
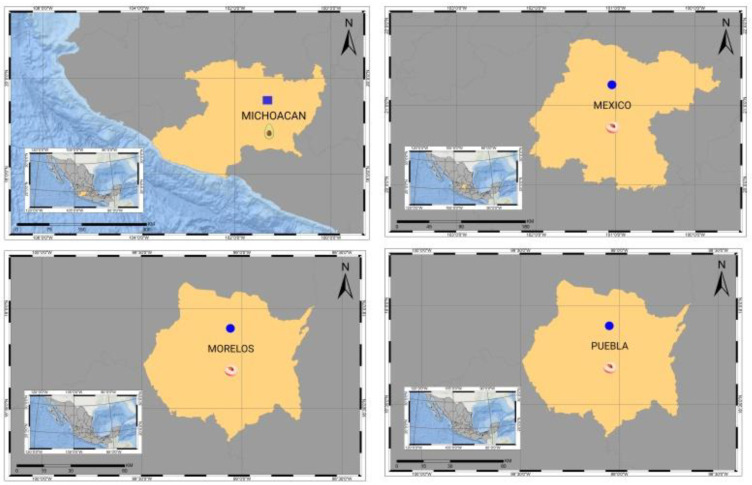
Distribution of viroids in Mexico associated with fruit-trees diseases. ASBVd-infected avocado trees in the state of Michoacan [17]. In the central region of Mexico, the presence of PLMVd was detected in peach orchards in the states of Puebla, Morelos, and Mexico [19].

Michoacan is the world leader in avocado production. During the period of July 2019 to June 2020, Mexico exported about 964,000 tons of avocado to the United States [60]. The United States is the main purchaser of Mexican avocados, monopolizing 90% of the exportable production. Currently, 51 of the 113 municipalities in the Michoacan state have avocado production in a total area of 140,000 hectares, of which 120,000 are certified [61]. Variants of the ASBVd were reported in avocado groves of Michoacan [18] Figure 3. The ASBVd isolates from Mexico have a 98.16% (LT966069.1) and 96.27% (HQ700589.1) nucleotide sequence identity to variants from Europe, Asia, and Oceania Figure 2b. Later the same research group published the first report on PLMVd in Mexico [19]. During the summer of 2013–2014, symptomatic plants of peach cultivars were identified in the States of Mexico, Morelos, and Puebla, Figure 3. In this report, assays were performed to identify the possible presence of PLMVd and HSVd. Both viroids infect peach naturally in other countries [62]. However, only PLMVd was detected in the Mexican samples. The PLMVd isolates were 99%–97% identical to the corresponding sequences of PLMVd reported in the NCBI for Montenegro, Australia, and South Korea, Figure 2b.

## 3. Genetic Variants of Pospiviroid Species Endemic to Mexico

TPMVd was the first viroid associated with the *Planta Macho* disease infecting tomato crops in Mexico [44]. However, later it was identified as Mexican papita viroid from *S. cardiophyllum* as a wild symptomless reservoir [12]. For that reason, it was proposed that this viroid could be the closest to the viroid’s ancestor [12]. MPVd, as the ancestral viroid, may have an evolutionary link with TCDVd in the development of crop diseases [63].

TPMVd and MPVd genetic variants are endemic species and have shared and unique, unusual features. Numerous questions as to their molecular and biological properties remain unanswered [64]. In 2011 Verhoeven et al. questioned earlier reports that indicated these viroids should be classified as different species based on plant models as biological indicators and proposed that they should be considered members of a single species [65]. Currently, both viroids belong to the TPMVd species since it was discovered approximately ten years earlier than MPVd.

**Figure 4:**
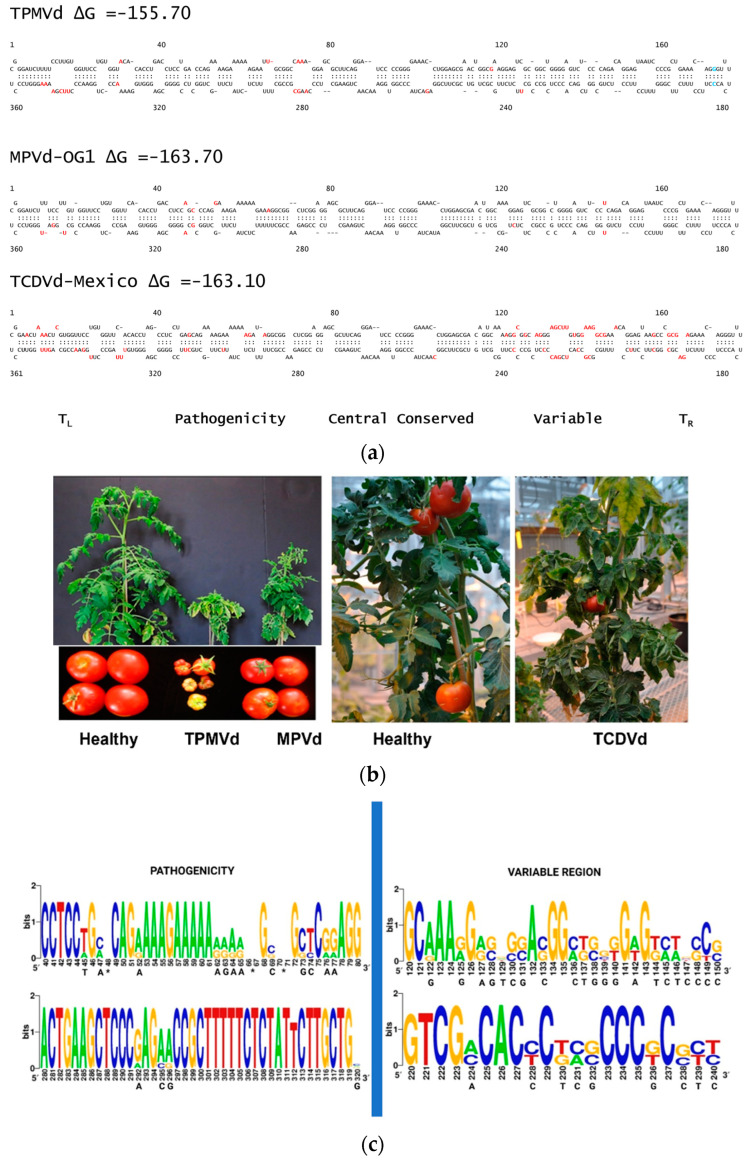
(**a**) Complete nucleotide sequences and secondary structures predicted using the UNAFold Web Server (UNAFold tool available online: www.mfold.org accessed on 15 May 2022) [69] for TPMVd (GenBank K00817), MPVd-OG1 (GenBank L78454), and TCDVd-Mexico (GenBank GQ131572). The nucleotide differences compared to a consensus sequence from alignment of the three viroids are shown in red. The single base pair in the T_R_ domain determining virulence in TPMVd is shown in blue [64]. The putative functional domains are as defined by Keese and Symons [70]; T_L_ = terminal left, T_R_ = terminal right. (**b**) Symptoms of viroid species inoculated onto cv. Rutgers tomato plants 8 wks post-infection. (**c**) Sequence logo depicting the alignments of the Pathogenicity (40–80 nt upper and 280–320 lower strands); and Variable domains (120–150 nt upper and 220–240 lower strands) of the complete genomes of TPMVd, MPVd, and TCDVd-Mexican viroid isolates. Uppercase letters in black color under the number of the nucleotide represent those that correspond to the TPMVd genome.

Regarding their secondary functional structure, a one base-pair mutation in the Terminal Right (TR) domain (176U: A185 in the mild variant and 174G: C183 in the severe variant) was identified as a virulence determinant factor in TPMVd [64]. Additionally, the Terminal Left (TL) and Pathogenicity domains of TPMVd are involved as major determinants for pollen horizontal and vertical transmission [66]. In Coleus blumei viroid 1, a loop in the TL region was identified as a key determinant for a high rate of seed transmission [67]; however, a similar motif has yet to be determined for pospiviroids. The TR domain forms a relatively independent structure that contributes to modulating the efficiency of viroid replication and accumulation [68], Figure 4a.

TPMVd is closely related phylogenetically to tomato apical stunt viroid (TASVd), TCDVd, CEVd, and PSTVd, the causative agent of spindle tuber disease of potato in a large number of countries [9,71,72,73]. Among the seven pospiviroid species that have been described as capable of replicating in a tomato host, TPMVd exhibits the most severe symptoms in susceptible cultivars, including severe stunting, epinasty, stem necrosis in some tomato cultivars, and tiny tomato fruits Figure 4b. The differences in the symptom development in the tomato host must be due to the specific nucleotides in particular viroid genome domains. A multiple sequence alignment of the complete viroid genomes corresponding to the Mexican isolates of TPMVd, MPVd, and TCDVd indicates that the most variable regions are the Pathogenicity (P) and Variable (V) domains, particularly the upper strand of the P domain when differences in the consensus sequence were compared. These findings correlate with previously reported hotspots of origin of viroid-derived small interfering RNAs, which have been recognized as pathogenic effectors [70]. In addition, the T_L_ domain of TCDVd differs markedly from the same domain in TPMVd and MPVd and may be a factor in explaining why seed transmission was not demonstrated for TCDVd, despite extensive efforts [63].

## 4. Tomato Crop Diseases: Role of *Solanaceae* Host Endemic Species and Putative Vectors

Notably, among TPMVd natural hosts, many species belong to the *Solanaceae* family. A wide range of wild species reservoirs, such as *S. nigrescens*, *S. rostratum*, *Physalis aff. foetens*, *Physalis philadelphica*, *Jalmota procumbens*, *S. cardiophyllum*, and agronomically essential crops such as *Capsicum annum* (Chili pepper) and *S. lycopersicum* (tomato) have been reported. Among those natural hosts, tomato susceptible varieties exhibit the most severe symptoms [10,12,41].

**Figure 5:**
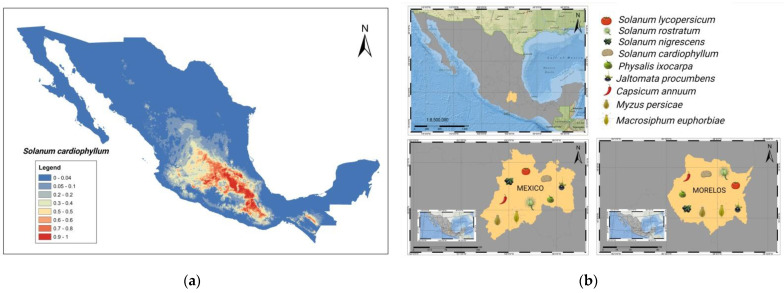
(**a**) Mexico’s central region shows the greatest distribution values according to the maximum entropy modeling (Maxent) for *Solanum cardiophylum* species, an endemic wild reservoir of viroids. (**b**) A remarkable distribution of the insects and the *Solanaceae* natural hosts are found in Mexico and Morelos States.

The central region of Mexico represents a geographical area with an abundant presence of those host endemic species. This observation agrees with the region where the *Planta Macho* disease was first described almost 50 years ago. Moreover, this area shows the maximum entropy values for the establishment of *S. cardiophylum* species, the first wild species reservoir of MPVd replication without symptoms, Figure 5.

Viroids are easily transmitted by mechanical inoculation [74] and efficiently dispersed by contact, for example, by tools used in pruning, clothing, handling the crop with hands, and direct contact between nearby plants [75,76]. Notwithstanding, some species can also be transmitted by seeds, vegetative propagation, grafting, pollen, and insects [77].

In addition, TPMVd can be transmitted through seeds, pollen, and aphids [78]. These features make it a highly transmittable viroid species. The EPPO considers TPMVd and other pospiviroids as quarantine pests [79].

It is noteworthy that TPMVd has a high rate of horizontal transmission by pollen, whereas other pospiviroids, such as PSTVd, do not [66]. In addition, TPMVd was the first species reported to be transmitted by an aphid vector. Earlier reports highlighted the role of vector aphid (*Myzus persicae*, peach aphid) in the transmission of TPMVd from the wild host *Physalis foetens* to the experimental host tomato [44,80]. Moreover, it was stated that the transmission efficiency relies upon the plant host.

*M. persicae* is a polyphagous species capable of feeding on 89 species of plants belonging to 43 families and 71 genera [80]. It is distributed worldwide and is considered the aphid species that can transmit the largest number of phytopathogenic viruses (more than 100). Additionally, many of these can be transmitted in a non-persistent and circulatory way.

In Mexico, the first records of aphids were in potato, pepper, broccoli, and cabbage [81]. The largest number of aphid host species belong to *Asteraceae* and *Solanaceae.* Among the species found in this family, the following *P. ixocarpa*, *P. leptophylla*, *P. philadelfica*, *S. cervantesii*, *S. diversifolium*, and *S. rostratum*, [80] and those that belong to the genera *Capsicum*, *Datura*, and *Nicotiana*, are all considered TPMVd natural hosts [82].

Earlier experiments were carried out to determine which of the seven most promising viroid natural hosts were the best source of inoculum for transmission through *M. persicae*. Among the most favorable were *P. ixocarpa*, with 97% transmission, and wild pepper, with 75% transmission, followed by *S. negrescens*, *S. rostratum*, and *Jaltomata procumbes*. Notably, the least favorable was the tomato host. In those experiments, all the instars and winged forms of the aphid were found capable of transmitting [83].

It was evident that there were significant differences in viroid transmission by the peach aphid when different hosts of the viroid were used as a source of inoculum. Tomato, which is not a good source of inoculum nor is it preferred by the aphid, does not seem to play an essential role in the epiphytiology of the disease. On the contrary, other wild reservoirs such as *P. ixocarpa*, *S. rostratum*, *or S. cardiophylum*, among others, could play an important role in this aspect, Figure 5b.

**Figure 6:**
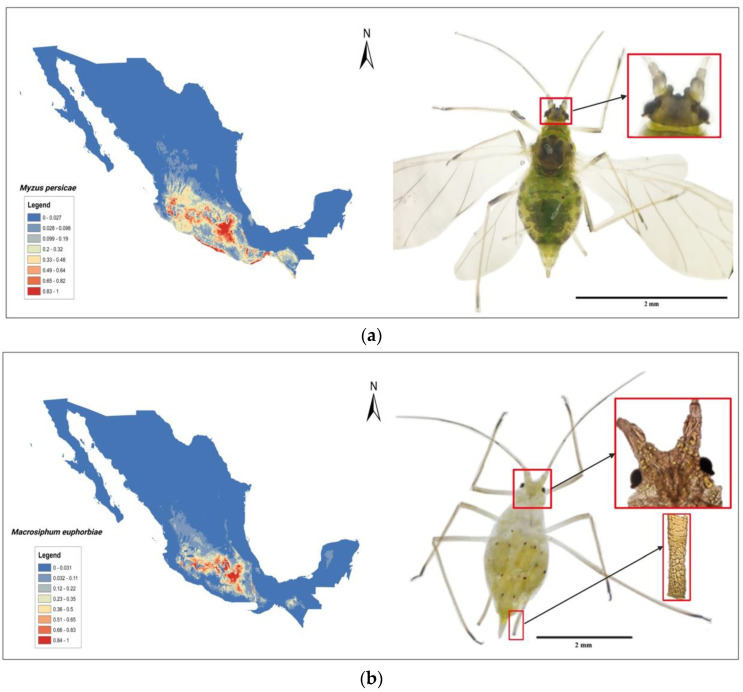
Geographical zones of greater adaptability according to maximum entropy modeling (Maxent); (**a**) of peach aphid, right: winged adult green peach aphid (*M. persicae*) is observed on the dorsum of the abdomen with a large dark patch and is framed by the antennal tubercles are well developed and convergent, and (**b**) of potato aphid, right: wingless female adult potato aphid (*Macrosiphum euphorbiae*) where the antennal tubercles are well developed, and the cornicle has an apical polygonal reticulation area.

In this context, the distribution and incidence of both of the viroid known vectors *M.persicae* and *Macrophibum euphorbiae* (potato aphid) are correlated with the geographic region in the Trans-Mexican Volcanic Belt where Morelos and Mexico are the principal states with the higher entropy values, Figure 6a 22 °C isothermal condition previously defined the TPMVd disease distribution. It has been reported that tomato fields with an annual mean temperature above 22 °C (800 to 1400 m altitude) had a disease incidence of up to 45%, while those with an annual mean temperature below 22 °C were free of the disease [10].

Indeed, the distribution of those wild endemic *Solanaceae* species and the presence of the insect vectors could play a key role in viroid biology and the origin of crop diseases. Although viroids are primarily transmitted mechanically, the transference *“jump”* between a natural wild host (not cultivable) to an economic crop could be due to insect vector transmission. Viroid transference from wild natural hosts to commercial tomato plants appears possible by an *M. persicae* biotype able to transmit the viroid, which could colonize the multiple viroid natural host species [7]. Nevertheless, the role of other insects, such as *Macrosiphum* spp., in transmission, possibly by feeding on wild reservoirs, cannot be ruled out, Figure 6b.

The complexity of the pathosystem between the insect and viroid transmission in natural hosts has been recently reviewed [76]. For example, PSTVd could be transmitted in a non-persistent manner by the potato aphid but could not be transmitted by *M. persicae* Sulzer [84,85]. In contrast, TCDVd can be transmitted by the peach aphid only in a mixed infection with potato leaf roll virus (PLRV) [86]. To add more to the complexity, apple chlorotic fruit spot viroid (ACFSVd) was recently detected in peach aphids collected from symptomatic trees, which suggests the aphid is a carrier but not a vector of this viroid species [87].

**Figure 7:**
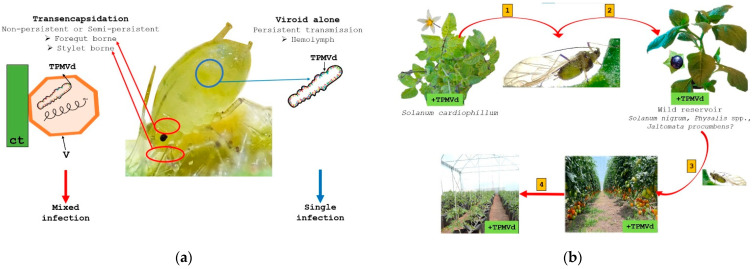
(**a**) Depicts the complexity underlying the aphid transmission mechanism. Aphid transmission could occur by two mechanisms: the viroid is *transencapsidated* into a virus (V) (ct = cuticle), or the vector aphid is capable of transferring the viroid by itself. (**b**) A putative model of viroid transference from a symptomless wild reservoir to a susceptible commercial tomato host. First, the aphid feeds from a viroid-infected wild reservoir; next, the aphid vector transmits the viroid to other solanaceous natural hosts; and finally, the vector spreads the infection to some tomato plants in crop fields. Viroid-infected cultivated plants disseminate the disease under greenhouse conditions by seeds, mechanical inoculation, or vegetative propagation.

Since viroids do not encode and are not encapsidated by any protein, it can be assumed that insect transmission is due only direct physical contact of infected sap with some part of the insect or through *transencapsidation*. *Transencapsidation* is a process defined as the encapsulation of the nucleic acid within the virion of another virus [88], Figure 7a. A putative model of viroid transference from a wild reservoir to a commercial tomato host is described in Figure 7b. Further observations on the presence or absence of hosts, parasites, crop conditions, and the environment will contribute to a better understanding of the biology of viroid diseases.

## 5. ASBVd and the Origin of Avocado Plants

The sunblotch disease was observed for the first time in southern California in 1914 and later in Palestine in 1924 [89]. However, the first official report of the disease was published in 1928 in the USA, and the symptoms were attributed to solar irradiation [90] and to a genetic disorder [91]. It was not until 1981 that ASBVd was confirmed as the causal agent of avocado sunblotch disease. At that time, healthy avocado trees developed symptoms of the disease when inoculated with the bark of infected trees and filter paper pieces moistened with the purified viroid. The infection by ASBVd was confirmed in seedlings using PAGE and cDNA probe methods [92]. To date, the most reliable method to detect ASBVd is the identification of symptomatic fruits and confirmation-sensitive molecular laboratory techniques to identify asymptomatic infected plants. These asymptomatic plants are essential in spreading this disease, and avocado nurseries must be certified to ensure pathogen-free seeds. However, it was unexpected that, in a recent analysis, symptomatic trees were negative by qRT-PCR (false negatives) (Ochoa-Martínez, personal observations). The development of new techniques, such as the computational predictions of disrupted metabolites in leaf tissue or the implementation of *“deep learning”* approaches, such as those reported for virus diagnosis [93], will aid disease detection in the analyzed samples.

ASBVd has a restricted host range composed mostly of plant species of the family *Lauraceae*. The avocado (*Persea americana* Mill.) belongs to the *Lauraceae* family, one of the oldest among flowering plants. Sunblotch disease symptoms are variable, in fruits are irregular sunken areas of a white, yellow, or reddish color, and the sunken areas may become necrotic. Some green shoots and branches sometimes show superficial stripes at the base, the leaves are deformed, and the leaf blade is reduced. Some infected trees are asymptomatic but may develop symptoms under stress conditions. Likewise, symptomatic trees may become asymptomatic for underexplored reasons. More research is needed to understand the distribution and possible variants associated with the different symptoms that have been identified [20].

ASBVd is distributed worldwide and is a quarantine pathogen since it causes significant reductions in yield and fruit quality. This viroid disease occurs in six continents: North and South America, Europa, Asia, Africa, and Australia, where avocados are grown [94]. The disease spread from California to Florida (1939), Venezuela (1976), Australia (1979), South Africa (1984), Spain (1987), Peru (1991), and later to Mexico (2009) [95]. In Mexico, the ASBVd has been reported in the Michoacan State, mainly confirmed areas of their presence are Tingambato and Uruapan municipalities [96].

Avocado production is primarily in geographical regions with a tropical or Mediterranean climate. Mexico is undoubtedly the reference country in terms of production regarding avocado production [97]. The name avocado comes from the Nahuatl *ahuacatl*, which means *“testicles of the tree”.* The center of origin of avocado plants is very possibly located in Mesoamerica (highlands of Mexico and Guatemala), particularly the cloud forests in the region of the Trans-Mexican Volcanic Belt of Mexico. The oldest avocado fossils, dated more than 8000 years ago and were found in the Caves of Coxcatlan in the Tehuacan Valley of the State of Puebla, Mexico [98].

Recently, it has been proposed that the origin of avocado groves is linked to the origin of ASBVd in the Mexican highlands [20]. In biological observations, it was noted that in symptomatic avocado fruits with ASBVd, the embryo generally atrophies, while in asymptomatic fruits with a lower viroid load, viable embryos are present. Moreover, the absence of ASBVd in some wild populations of Mexican avocado (*P. americana* var. drymifolia) was observed. Therefore, it will be interesting to identify the geographical location of viroid-infected wild hosts. Due to their economic impact, the presence of ASBVd has only been confirmed in some commercial orchards.

## 6. Citrus Exocortis and Other Citrus Viroid Species

**Figure 8:**
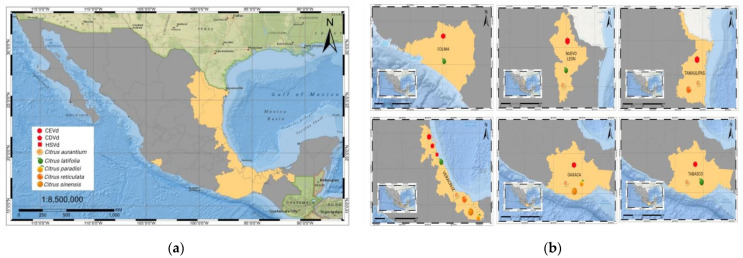
(**a**) Distribution of viroids in Mexico associated with citrus diseases. (**b**) Geographical areas and description of the affected citrus plantations by the different viroid species.

CEVd, CDVd, and HSVd have been reported to affect multiple citrus hosts. CEVd has been reported to infect at least 50% of the citrus in the northeast region (Tamaulipas and Nuevo Leon) Figure 8. Through bioassays using the indicator plant Etrog citron, *Citrus medica*, CEVd has been isolated from sweet orange *Citrus sinensis* cv. Osbeck, *Citrus paradisi* Macf., mandarin orange *Citrus reticulate* Blanco, and mandarin hybrid trees [15,28]. The effect of CEVd on citrus production may sometimes be overlooked because of the absence of symptoms in trees grafted on the sour orange rootstock *Citrus aurantium*, which is in general use throughout the country.

In contrast to the CEVd infection of citrus described above, CEVd infection of *Tahiti* (Persian) lime, *Citrus latifolia*, which is also a profitable citrus crop in Mexico, is more consequential. *Tahiti* lime has a production of around 20,000 ha, while Mexican lime has a production of ~ 80,000 ha. Mexico is the principal producer of acid limes worldwide, primarily for export and consumption as fresh fruit in local markets. CEVd exhibits severe symptoms of cracking in branches in almost all the producer regions of Mexico (Colima, Tabasco, and Veracruz), leading to low yields in the plantations and reduced longevity of the groves. Other states reporting CEVd infection in citrus are Nuevo Leon, Oaxaca, and Tamaulipas, Figure 8b [15,16,99,100].

Tahiti lime has traditionally been grafted onto the sour orange rootstock. However, many new groves have been planted on Troyer, Carrizo citrange, Volkamer lemon, and Alemow rootstocks. Tahiti lime grafted onto Alemow *C. macrophylla* Webster rootstock infected by viroids [15,16,101,102] often shows symptoms such as reduced tree size with brown lesions in the wood just below the site of the graft [103], which are typical symptoms reported for the disease known as cachexia [28,99]. It has been demonstrated that citrus cachexia is caused by the viroids citrus viroid IIb (CVdIIb) or CVdIIc, variants of the HSVd [99]. Recent reports have shown the incidence of cachexia in *Citrus latifolia* in Veracruz, Mexico [100].

A recent study has pointed out that CEVd and HSVd were detected in severe symptomatic and asymptomatic orange trees in Veracruz. Notably, neither viroid presence was found in plants with mild symptoms. The difference in symptom development was related to the coexistence of severe CTV strain and both viroid species in these plants. The presence in asymptomatic plants and the coexistence of these viroids only with the severe but not with the mild CTV strain suggest that they may be selectively widespread in orange and other citrus species. Further bioassays with severe and mild CTV isolates and CEVd or HSVd would reveal their complex role in disease development.

## 7. Emerging Diseases

**Figure 9:**
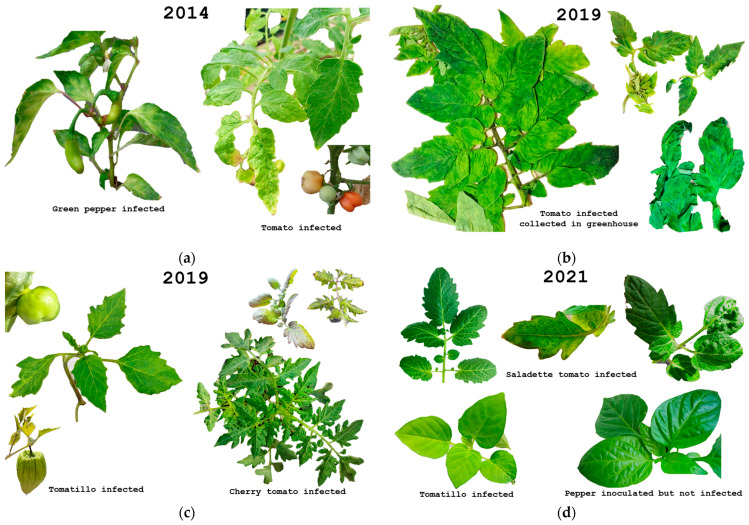
Follow-up study of MPVd isolated from high-tech greenhouses in central Mexico. (**a**) Tomato and green pepper inoculated during 2014 with MPVd showing severe chlorosis and deformed leaves and small and deformed fruits. (**b**) Tomato MPVd-positive plants with chlorotic and deformed leaves collected in 2019 in greenhouse. (**c**) Tomatillo and tomato cv cherry inoculated in 2019 with MPVd; in tomatillo, chlorotic and deformed leaves and fruits were observed; in cherry tomato, chlorotic and purple leaves were observed. (**d**). Tomato cv Saladette, tomatillo, and pepper inoculated in 2021 with MPVd. Saladette showed chlorotic and some deformed leaves, tomatillo only showed slightly chlorotic leaves, and pepper was not infected and no symptoms were observed.

Interestingly, MPVd has been continuously reported to infect tomato plants under greenhouse conditions, causing severe to mild symptoms [104]. From 2013 to 2019, MPVd was detected in high-technology tomato greenhouses in the state of Mexico, Figure 9. MPVd-infected tomato, pepper, and tomatillo plants were tested in bioassays. In those experiments, *S. lycopersicum* var. saladette and *C. annum* plants were infected under greenhouse conditions, and the most severe symptoms of this follow-up study were identified in those host plants from 2014 to 2016. The symptoms observed were severe chlorosis, stunting, and a purple hue in mature leaves. Subsequently, the fruits that could be harvested were deformed and significantly smaller when compared to those of mock-inoculated plants, Figure 9a.

In 2017, MPVd-infected plants were obtained from the same high-tech greenhouse. Notwithstanding, when the biological assays were carried out following the same experimental conditions, for unknown reasons, it was impossible to systemically infect the model tomato plants (negative by RT-PCR). Subsequently, from 2018 to 2019, viroid-positive tomato plants were obtained again from the same greenhouse, and from 2019 to 2021, the experiments showed, in contrast with previous observations, only slight symptoms in susceptible test plant models, including tomato cherry, tomato cv saladette, and tomatillo, Figure 9b. In 2019 when we inoculated tomato cv saladette with symptomatic tissue, the only symptom observed was chlorosis, while in the cherry variety more severe mosaic turning purple when the leaves matured was observed, along with growth reduction, Figure 9c. In the same year, only half of the inoculated tomatillo plants showed leaf mosaics and deformed leaves and fruits symptoms, Figure 9c. In 2021, the symptoms on the saladette variety were chlorosis and, in only some plants, deformed leaves, Figure 9d. Meanwhile, in all tomatillo plants inoculated, only slight chlorosis was identified, Figure 9d. Surprisingly, no symptoms or viroid replication were observed in pepper plants inoculated during the experiments carried out, Figure 9d.

Of considerable interest, in 2018, MPVd was persistently detected in greenhouses, in mixed infections with tomato severe leaf curl virus (ToSLCV), a begomovirus species [101]. Tomato plants with mixed infections displayed severe symptoms.

However, with the high persistent replication of MPVd isolates under greenhouse conditions, the disease does not represent a considerable threat to greenhouse tomato production because of its mild symptoms. Since 2019, detections have been focused on identifying tomato brown rugose fruit virus, a highly severe emerging disease in the tomato industry.

## 8. Perspectives

It is currently unknown if TPMVd/MPVd or other viroid species are spreading and evolving in the central region of Mexico. Moreover, the presence of other viroid species in Mexico cannot be ruled out, given studies reporting the high adaptability of these RNA pathogens to new hosts. For example, PSTVd (absent in Mexico), which was long considered to infect only potato crops in the field, has been reported to infect tomato and avocado plantations. Moreover, ASBVd has been known to be vertically transmitted. Therefore, there is potential for a viroid to invade new hosts if conditions allow [102,103,104,105]. In addition, the existence of latent infections may play an important role in the transfer and widespread contamination of susceptible plants, resulting in disease.

Regarding the pivotal biological role of insect vectors in viroid disease epidemiology, we must consider that earlier experiments employed techniques relying upon plant bioassays to confirm that viroids can be transmitted directly by aphids, apparently without mixtures of infections with other viruses. The confirmations were based only on the appearance of symptoms or on the inoculation of indicator plants. At that time, the molecular and sequencing techniques that would confirm the detection of the viroid in the aphid and rule out mixtures of infections with other viruses were unavailable. Therefore, we were interested in further examining the role of *M. persicae* and *M. euphorbiae* aphids in viroid transmission.

We mechanically inoculated tomato, wild tomato, and pepper using tomato plants infected with TPMVd (MPVd) collected in greenhouses in the state of Mexico as inoculum. Symptomatic and mock-inoculated plants were used during 2018 and 2019, at least in three different transmission experiments with the potato aphid. Our results showed that it was not possible to detect the presence of the viroid by RT-PCR in either the aphid fed on viroid-infected plants or in the receptor plants by RT-PCR. Transmission experiments are currently being carried out with the peach aphid using different infected natural wild hosts.

Adding to the complexity of viroid diseases, there is little understanding of the role of virus/viroid coexistence in mixed infections, and more study is warranted. To date, at least three mixed viroid infections have been reported in Mexico: MPVd/TCDVd and MPVd/ToSLCV in tomato under greenhouse conditions, and HSVd/CEVd, CTV in orange trees. Symptoms of mixed infected plants were strongest than those of single infections in all cases.

As we are aware of the high complexity of viroid disease etiology, our current efforts are focused on employing high throughput sequencing (HTS) technologies to determine the differences and possible conserved mutations underlying the evolution and biodiversity of endemic strains, as well as to obtain deep insight into the role of mixed infections and insects in their transmission.

Understanding their biology and epidemiology is essential to developing effective measures to defeat viroid diseases. In current plant pathology studies, it is pivotal to elucidate how a pathogen has evolved to create mechanisms crucial to favor its survival to manage emerging diseases effectively. Studies on these issues will be vital to producing technological tools to help combat the symptoms expressed in the plant that generate significant economic losses worldwide. With the scenario of viroid pathogenesis being considerably more complex than previously thought and with the emergence of viroid diseases that can lead to high financial losses in the agricultural industry that they represent, future studies will be helpful for programs to limit movement and spread to avoid economic losses in agricultural industries.

## Figures and Tables

**Table 1:** Economic effects of viroid species reported in Mexico, mode of spread, and control strategy.

Family	Species ^a^	Economic Host/Disease	Damage	Cost	Mode of Spread ^b^	Control Strategy
*Pospiviroidae*	CDVd	Citrus groves	Variable	Reduced yield	Vegetative: +	Eradication viroid-free
					Seed/pollen: −	
					Insect: −	
					Mechanical: +	
	CEVd	Dwarfing	Variable	Reduced yield	Vegetative: +	Eradication viroid-free
					Seed/pollen: +/−	
					Insect: −	
					Mechanical: +	
	HSVd	Exocortis	Variable	Reduced yield	Vegetative: +	Eradication viroid-free
		Cachexia			Seed/pollen: +/−	Propagation material
					Insect: −	
					Mechanical: +	
	MPVd	Tomato	Severe	Yield	Vegetative: NA	Eradication
		Greenhouses			Seed/pollen: NK	
		Dwarfing			Insect: NK	
		Chlorosis			Mechanical: +	
	TCDVd	Tomato	Severe	Yield	Vegetative: NA	Eradication
		Greenhouse			Seed/pollen: +/−	
		Dwarfing			Insect: +	
		Chlorosis			Mechanical: +	
	TPMVd	Tomate field	Severe	Yield	Vegetative:NA	Eradication
		Planta macho			Seed/pollen: +	
					Insect: +	
					Mechanical: +	
*Avsunviroidae*	ASBVd	Avocado groves	Severe	Reduced yield	Vegetative: +	Eradication viroid-free
		Sunblotch		Discarded fruit	Seed/pollen: +	Propagation material
					Insect: −	
					Mechanical: +	
	PLMVd	Peach orchards	Variable		Vegetative: +	Propagation material
					Seed/pollen: +	
					Insect: +	
					Mechanical: +	

^a^ citrus dwarfing viroid (CDVd), citrus exocortis viroid (CEVd), hop stunt viroid (HSVd), Mexican papita viroid (MPVd), tomato chlorotic dwarf viroid (TCDVd), tomato planta macho viroid (TPMVd), avocado sunblotch viroid (ASBVd), peach latent mosaic viroid (PLMVd). ^b^ NA = Not applicable; NK = Not known.

**Table 2:** Viroids species reported in Mexico and their worldwide distribution.

Family	Species ^a^	Distribution in Mexico	Other Countries	Detection ^b^	Reports
*Pospiviroidae*	CDVd	Veracruz	Uruguay	2016 ^1^, 2002 ^2,3^	[21,22]
		Nuevo León	USA	2013 ^1^, 2020 ^2,3^	[23,24]
		Tamaulipas	Egypt	2013 ^1^, 2017 ^2,3^	[23,25]
		Mexico	Italy	2013 ^1^, 2013 ^2,3^	[23,26]
			Laos	2020 ^2,3^	[27]
	CEVd	Nuevo León	China	1979 ^1^, 2013 ^1^, 2012 ^2,3^	[23,28,29]
		Tamaulipas	Tunisia	1995 ^1^, 2013 ^1^, 2014 ^2,3^	[15,23,30]
		Veracruz	Iran	1999^1^, 2018^1^,2017 ^2,3^	[13,31,32]
		Tabasco	USA	1999 ^1^,2000 ^1^, 2021 ^2,3^	[13,33,34]
		Mexico		2013 ^1^	[23]
	HSVd	Nuevo León	China	2013 ^1^, 2015 ^2,3^	[23,35]
		Tamaulipas	Italy	2013 ^1^, 2021 ^2,3^	[23,36]
		Mexico	Laos	2013 ^1^, 2020 ^2,3^	[23,27]
		Veracruz	Turkey	1999^1^,2018 ^1^, 2013^2,3^	[13,31,37]
		Tabasco		1999 ^1^	[38]
	MPVd	Aguascalientes	Canada	1996 ^1^, 2010 ^2,3^	[12]
		Mexico City		2009 ^1^	[39]
	TCDVd	Mexico City	France	2009 ^1^, 2010 ^2,3^	[39,40]
		Jalisco	Belgium	2011 ^1^, 2016 ^2,3^	[41,42]
			Japan	2009 ^2,3^	[43]
	TPMVd	Mexico	Canada	1974 ^1^, 2010 ^2^	[38,44]
		Morelos		1978 ^1^	[8]
*Avsunviroidae*	ASBVd	Michoacán	Australia	2009 ^1^, 2011 ^2,3^	[18,45]
			Greece	2018 ^2,3^	[46]
			Israel	2013 ^2,3^	[47]
	PLMVd	Mexico	South Korea	2013 ^1^, 2018 ^2,3^	[19,48]
		Puebla	Montenegro	2014 ^1^, 2011 ^2,3^	[19,49]
		Morelos	Australia	1978 ^1^,2012 ^2,3^	[19,50]

^a^ Same acronym abbreviations as defined in Table 1. ^b1^ Reports in Mexico, ^b2^ Reports in other countries,^b3^ Isolates with 85% of homology to Mexican strains source: NCBI (www.ncbi.nlm.nih.gov/ accesed on 3 June 2022).

## Data Availability

Not applicable.
